# Upfront surgery and pathological stage-based adjuvant chemoradiation strategy in locally advanced esophageal squamous cell carcinoma

**DOI:** 10.1038/s41598-018-20654-0

**Published:** 2018-02-01

**Authors:** Hui-Shan Chen, Po-Kuei Hsu, Chia-Chuan Liu, Shiao-Chi Wu

**Affiliations:** 1 Department of Health Care Administration, College of Health Sciences, Chang Jung Christian University, Tainan, Taiwan; 2 Division of Thoracic Surgery, Department of Surgery, Taipei Veterans General Hospital and School of Medicine, National Yang-Ming University, Taipei, Taiwan; 3 Division of Thoracic Surgery, Department of Surgery, Koo Foundation Sun Yat-Sen Cancer Center, Taipei, Taiwan; 4 Institute of Health and Welfare Policy, National Yang-Ming University, Taipei, Taiwan

## Abstract

Adjuvant chemoradiation is reported to have a survival benefit for esophageal squamous cell carcinoma (ESCC). We evaluated the “upfront surgery and pathological stage-based adjuvant chemoradiation” strategy, in which adjuvant therapy is guided by pathological stage, in locally advanced ESCC. Data from 2976 clinical stage II/III ESCC patients, including 1735 in neoadjuvant chemoradiation and 1241 in upfront surgery groups, were obtained from a nationwide database. Patients in the upfront surgery group were further categorized into the “upfront surgery and pathological stage-based adjuvant chemoradiation” and “upfront surgery only” groups. The 3-year overall survival (OS) rates in the “neoadjuvant chemoradiation”, “upfront surgery and pathological stage-based adjuvant chemoradiation”, and “upfront surgery only” groups were 41.5%, 45.8%, and 28.5%, respectively. In propensity score matched patients, the 3-year OS rate was 41.7% in the neoadjuvant chemoradiation group, compared to 35.6% in the “upfront surgery and pathological stage-based adjuvant chemoradiation” group (*p* = 0.147), and 20.3% in the “upfront surgery only” group (*p* < 0.001). No survival difference was observed between the “neoadjuvant chemoradiation followed by surgery” protocol and the “upfront surgery and pathological stage-based adjuvant chemoradiation” strategy.

## Introduction

Multidisciplinary therapy comprising surgery, chemotherapy, and radiotherapy is currently widely introduced in the treatment of esophageal cancer in an attempt to improve prognosis^1,2^. The strategy of neoadjuvant chemoradiation followed by surgery has been well recognized as an efficient approach. In the Chemoradiotherapy for Oesophageal Cancer Followed by Surgery Study (CROSS), the survival of patients with clinical stage T1N1 or T2-3N0-1 could be enhanced with the implementation of neoadjuvant chemoradiotherapy compared to surgery alone^3^. On the other hand, the strategy of upfront surgery followed by adjuvant treatment guided by pathologic findings has also been proposed by several retrospective studies. For adenocarcinoma, Zahoor *et al*. reported that upfront minimally invasive esophagectomy may be a reasonable approach, improving stage-based prognostication and potentially minimizing overtreatment in patients with clinical stage II or higher tumors^4^. For squamous cell carcinoma, Matsuda *et al*. reported that no significant survival difference was observed between “neoadjuvant chemotherapy” and “upfront surgery plus adjuvant chemotherapy for pN + stage” approaches in patients with clinical stage III tumors^5^.

We have previously evaluated the role of adjuvant chemoradiation in esophageal squamous cell carcinoma (ESCC) and found that surgery followed by adjuvant chemoradiation is significantly more effective than surgery alone at increasing the overall survival and decreasing recurrence rates^6,7^. Moreover, in patients who completed trimodal treatments, which included surgery and chemoradiotherapy, there was no difference in overall survival or disease-free survival between neoadjuvant (preoperative) and adjuvant (postoperative) chemoradiation groups^8^. Supported by these data, we aimed to evaluate the “upfront surgery and pathological stage-based adjuvant chemoradiation” strategy and compare it to the “neoadjuvant chemoradiation followed by surgery” approach in locally advanced ESCC. We hypothesized that upfront surgery can avoid unnecessary chemoradiation in patients who are clinically overstaged and that the outcome after adjuvant chemoradiation can be comparable to that after neoadjuvant chemoradiation.

## Results

### Analysis of the study population

A total of 2976 patients were included in this study (Fig. S1). The clinical characteristics of patients in neoadjuvant chemoradiation followed by surgical resection (group A) and upfront esophagectomy (alone or with adjuvant chemoradiation, group B) are summarized in Table 1. Patients who received neoadjuvant chemoradiation had more clinical stage III, cT3/4, and cN(+) tumors, longer tumor length, but lower age and Charlson’s comorbidity index (CCI). With regard to the surgical results (Table 2), 556 (32.1%) patients in group A had no detectable primary tumor at the time of resection, and 474 (27.3%) patients achieved complete pathological response (ypT0N0). On the other hand, the majority of the patients in group B had pT3/4 stage tumors (55.3%), and nearly half had pN + tumors (47.1%). The non-R0 resection rate was also higher in group B (13.1%) compared to group A (8.5%).

**Table 1:** Clinical characteristics before and after propensity score matching.

	Before matching			After matching		
	Group A	Group B	*p*	Group A	Group B	*p*
Total	1735	1241		562	562	
Age, years, mean ± SD	54.3 ± 8.7	56.0 ± 10.1	<0.001	54.6 ± 8.8	55.1 ± 10.0	0.384
Sex (%)			0.033			0.612
Male	1653(95.3)	1160(93.5)		531(94.5)	527(93.8)	
Female	82(4.7)	81(6.5)		31(5.5)	35(6.2)	
cStage (%)			<0.001			0.650
II	287(16.5)	728(58.7)		174(31.0)	167(29.7)	
III	1448(83.5)	513(41.3)		388(69.0)	395(70.3)	
cT stage (%)			<0.001			0.215
1/2	206(11.8)	557(44.9)		128(22.8)	111(19.8)	
3/4	1529(88.1)	684(55.1)		434(77.2)	451(80.3)	
cN stage (%)			<0.001			0.884
0	204(11.8)	510(41.1)		119(21.2)	121(21.5)	
+	1531(88.2)	731(58.9)		443(78.8)	441(78.5)	
Tumor length, cm, mean ± SD	5.9 ± 2.6	4.1 ± 2.2	<0.001	5.0 ± 2.4	5.1 ± 2.4	0.630
Location (%)			<0.001			0.820
Upper	243(14.0)	147(11.9)		75(13.4)	78(13.9)	
Middle	681(39.3)	454(36.6)		222(39.5)	208(37.0)	
Lower	396(22.8)	387(31.2)		146(26.0)	147(26.2)	
Unknown	415(23.9)	253(20.4)		119(21.2)	129(23.0)	
Differentiation (%)			<0.001			0.952
Well	36(2.1)	35(2.8)		20(3.6)	17(3.0)	
Moderate	821(47.3)	782(63.0)		345(61.4)	346(61.6)	
Poor	344(19.8)	326(26.3)		145(25.8)	149(26.5)	
Unknown	534(30.8)	98(7.9)		52(9.3)	50(8.9)	
CCI (%)			<0.001			0.279
0	1175(67.7)	695(56.0)		365(65.0)	345(61.4)	
1	393(22.7)	352(28.4)		126(22.2)	149(26.5)	
≥2	167(9.6)	194(15.6)		71(12.6)	68(12.1)	

Group A: neoadjuvant chemoradiation followed by surgical resection; Group B: upfront esophagectomy; SD: standard deviation; CCI: Charlson’s comorbidity index.

**Table 2:** Pathological results before and after propensity score matching.

	Before matching		After matching	
	Group A	Group B	Group A	Group B
Total	1735	1241	562	562
p (or yp) T stage (%)				
T0	556(32.1)	—	173(30.8)	—
Tis	31(1.8)	42(3.4)	10(1.8)	16(2.9)
T1	210(12.1)	287 (23.1)	77(13.7)	60(10.7)
T2	349(20.1)	222(17.9)	112(19.9)	78(13.9)
T3	514(29.6)	626(50.4)	173(30.8)	363(64.6)
T4	59(3.4)	61(4.9)	17(3.0)	45(8.0)
Unknown	16(0.9)	3(0.2)		
p (or yp) N stage (%)				
N0	1172(67.6)	616(49.6)	403(71.7)	225(40.0)
N1	380(21.9)	340(27.4)	107(19.0)	172(30.6)
N2	116(6.7)	197(15.9)	34(6.1)	120(21.4)
N3	41(2.4)	47(2.8)	9(1.6)	34(6.1)
Unknown*	26(1.5)	41(3.3)	9(1.6)	11(2.0)
Margin status (%)^†^				
Negative (R0)	1574(90.7)	1064(85.7)	512(91.1)	458(81.5)
Positive (R1/2)	148(8.5)	163(13.1)	48(8.5)	98(17.4)
Unknown	13(0.8)	14(1.1)	2(0.4)	6(1.1)

Group A: neoadjuvant chemoradiation followed by surgical resection; Group B: upfront esophagectomy;

*Definite positive lymph node number not recorded. ^†^*P* < 0.001 in both before and after matching groups.

**Figure 1:**
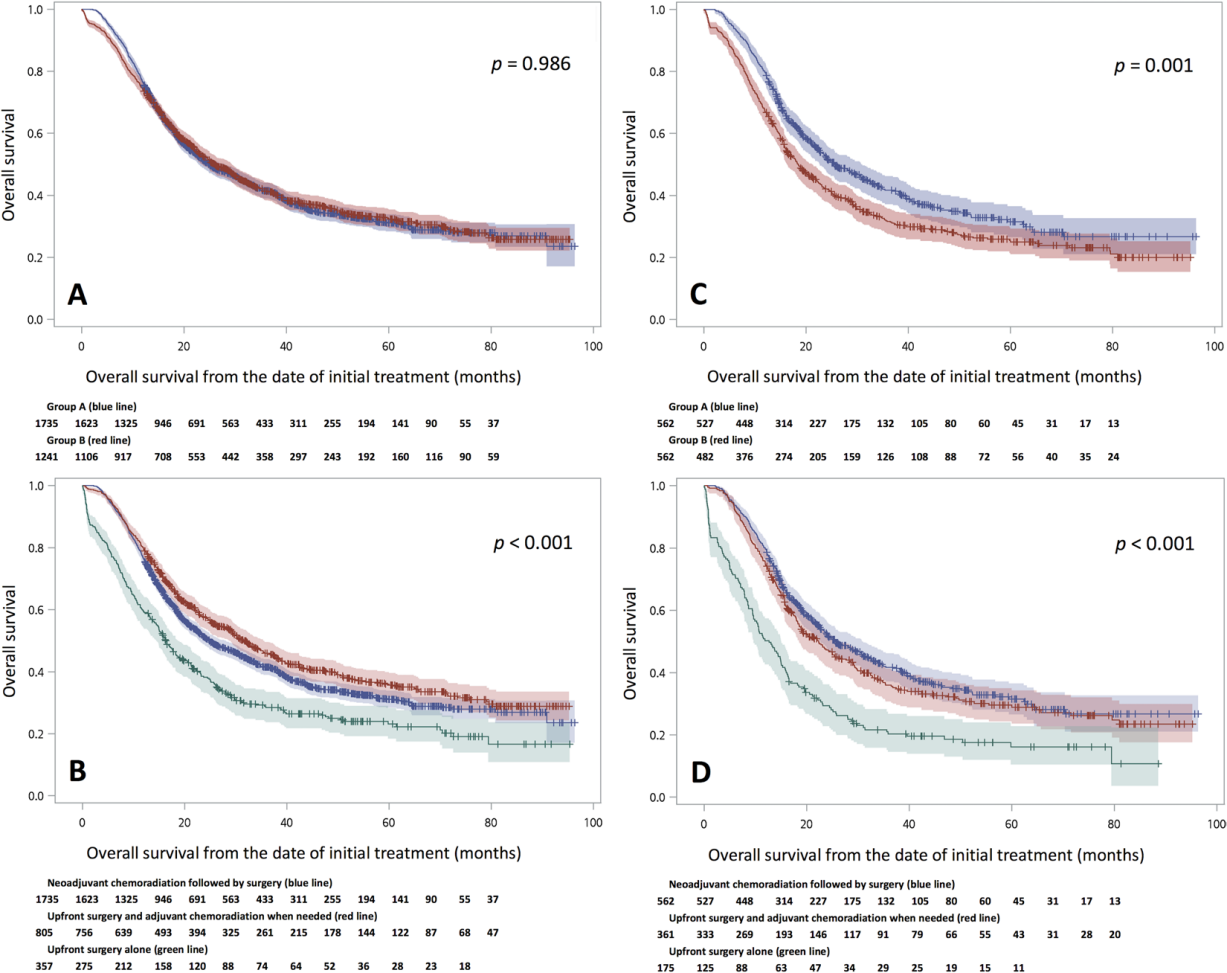
(**A**) Overall survival (and 95% confidence interval (CI)) of patients in group A (blue line) and group B (red line) groups, respectively. (**B**) Overall survival (and 95%CI) of patients in group A (blue line), “upfront surgery and pathological stage-based adjuvant chemoradiation” (red line), and “upfront surgery only” groups (green line). (**C**) Overall survival (and 95% CI) of patients in group A (blue line) and group B (red line) groups, respectively, in propensity score matched patients. (**D**) Overall survival (and 95% CI) of patients in group A (blue line), “upfront surgery and pathological stage-based adjuvant chemoradiation” (red line), and “upfront surgery only” groups (green line) in propensity score matched patients.

In the survival analysis, the median follow-up time was 37.1 (95% confidence interval (CI): 21.8–82.5) months for the surviving patients. There was no difference in overall survival between groups A and B. The 3-year overall survival rates and median survival were 41.5% and 24.8 (95%CI: 22.9–28.1) months in group A, versus 41.2% and 26.0 (95%CI: 23.5–29.3) months in group B (*p* = 0.986, Fig. 1A). According to the treatment modality, the patients in group B with complete pathological staging data (n = 1162) were categorized into the “upfront surgery and pathological stage-based adjuvant chemoradiation” and “upfront surgery only” groups. The former included patients receiving no further treatments for pathological T1-2N0 tumors and patients receiving adjuvant chemoradiation for pathological stage higher than T1-2N0 tumors, supported by our previous results that patients with pathological stages higher than T1-2N0 would benefit from adjuvant chemoradiation. In contrast, patients with pathological stage higher than T1-2N0 but received no further treatments were considered the “upfront surgery only” group. The 3-year overall survival rates and median overall survival in the “upfront surgery and pathological stage-based adjuvant chemoradiation” group were 45.8% and 31.5 (95%CI: 28.7–35.4) months, respectively, which was better than those in group A (*p* = 0.010) and those in the “upfront surgery only” group (28.5% and 16.4 (95%CI: 14.5–18.7) months, respectively, *p* < 0.001, Fig. 1B).

In the Cox regression model (Table 3), the significant prognostic factors in the univariable analysis included age, gender, cStage III, cT 3/4 stage, cN + stage, upper third location, poor differentiation, tumor length, higher CCI, non-R0 resection, and treatment strategy. In the multivariable analysis, two models with different definitions of treatment strategy were performed. Clinical stage III, cT 3/4 stage, poor differentiation, tumor length, higher CCI, and non-R0 resection remained independent factors in both models. The upfront surgery strategy, compared to neoadjuvant chemoradiation followed by surgery strategy, was an independent factor (hazard ratio (HR): 1.28; 95%CI: 1.09–1.49, *p* = 0.002) for worse overall survival. In contrast, the “upfront surgery and pathological stage-based adjuvant chemoradiation” strategy was not a significant prognostic factor (HR: 1.12; 95%CI: 0.94–1.33, *p* = 0.195) compared to the “neoadjuvant chemoradiation followed by surgery” group.

**Table 3:** Cox regression analysis for overall survival.

	Univariable analysis		Multivariable analysis			
			Model 1		Model 2	
	HR (95%CI)	*p*	HR (95%CI)	*p*	HR (95%CI)	*p*
Age (years)	1.01 (1.00–1.01)	0.006	1.00 (1.00–1.01)	0.505	1.00 (0.99–1.01)	0.958
Sex						
Male	1		1		1	
Female	0.75 (0.60–0.94)	0.010	0.77 (0.57–1.04)	0.086	0.69 (0.51–0.94)	0.019
cStage						
II	1		1		1	
III	1.43 (1.29–1.58)	<0.001	1.43 (1.10–1.86)	0.007	1.51 (1.16–1.98)	0.002
cT stage						
T1/2	1		1		1	
T3/4	1.53 (1.37–1.72)	<0.001	1.39 (1.14–1.70)	0.001	1.25 (1.02–1.54)	0.033
cN stage						
N0	1		1		1	
N+	1.20 (1.07–1.34)	<0.001	0.91 (0.72–1.14)	0.405	0.93 (0.73–1.18)	0.537
Location						
Lower third	1		1		1	
Middle third	0.96 (0.85–1.08)	0.478	0.94 (0.81–1.08)	0.378	0.96 (0.83–1.11)	0.585
Upper third	1.20 (1.03–1.40)	0.021	0.95 (0.78–1.17)	0.642	1.00 (0.81–1.22)	0.957
Differentiation						
Good/Moderate	1		1		1	
Poor	1.26 (1.12–1.41)	<0.001	1.31 (1.13–1.52)	<0.001	1.36 (1.17–1.58)	<0.001
Tumor length (cm)	1.01 (1.00–1.01)	<0.001	1.00 (1.00–1.01)	0.008	1.00 (1.00–1.01)	0.015
CCI	1.08 (1.03–1.13)	<0.001	1.14 (1.07–1.21)	<0.001	1.13 (1.06–1.21)	<0.001
Treatment strategy 1						
Group A	1		1			
Group B	1.001 (0.91–1.10)	0.986	1.28 (1.09–1.49)	0.002		
Treatment strategy 2						
Group A	1				1	
Upfront surgery and pStage based adjuvant CRT	0.87 (0.78–0.97)	0.013			1.12 (0.94–1.33)	0.195
Upfront surgery only	1.50 (1.31–1.72)	<0.001			1.86 (1.52–2.27)	<0.001
Margin status						
Negative (R0)	1		1		1	
Positive (R1/2)	2.53 (2.22–2.89)	<0.001	2.21 (1.84–2.65)	<0.001	2.31 (1.91–2.78)	<0.001

HR: hazard ratio; CI: confidence interval; CCI: Charlson’s comorbidity index; Group A: neoadjuvant chemoradiation followed by surgical resection; Group B: upfront esophagectomy; CRT: chemoradiotherapy.

### Analysis of propensity score matched patients

To decrease confounding effects due to nonrandomized assignment, propensity score matching was performed to identify 562 well-balanced pairs of patients whose clinical and pathological characteristics are shown in Tables 1 and 2, respectively. In the survival analysis, the 3-year overall survival rates and median overall survival were 41.7% and 25.6 (95%CI: 22.7–20.8) months in the neoadjuvant chemoradiation followed by surgery group, respectively, compared to 31.4% and 18.5 (95%CI: 16.3–21.3) months in the upfront surgery group (*p* < 0.001, Fig. 1C). However, when the patients in the upfront surgery group and with complete pathological staging data (n = 536) were further categorized, there was no survival difference (*p* = 0.147) between the neoadjuvant chemoradiation followed by surgery group and the “upfront surgery and pathological stage-based adjuvant chemoradiation” group, with 3-year overall survival rates and median overall survival of 35.6% and 22.3 (95%CI: 18.5–26.3) months. In contrast, the upfront surgery only group had the worst overall survival, with 3-year overall survival rates and median overall survival of 20.3% and 12.7 (95%CI: 9.7–15.1) months (Fig. 1D).

The patient characteristics of the “upfront surgery and pathological stage-based adjuvant chemoradiation” group and the “upfront surgery only” group are shown in Table 4. The patients in the upfront surgery only group had more pT3/4 tumors (89.2% vs. 68.1%, *p* < 0.001) and larger tumor lengths (5.4 ± 2.1 vs. 4.9 ± 2.5 cm, *p* = 0.001). However, higher age (57.1 ± 10.9 vs. 54.1 ± 9.2, *p* = 0.002) and CCI (≥1: 49.7% vs. 34.1%, *p* = 0.002) in the upfront surgery only group might be the reasons that interfered with the decision for adjuvant chemoradiation.

**Table 4:** Patient characteristics of upfront surgery and pathological stage-based adjuvant chemoradiation group and upfront surgery only group in the analysis of propensity score matched patients.

	Upfront surgery + pStage based adjuvant CRT	Upfront surgery only	*p*
Total	361	175	
Age, years, mean ± SD	54.1 ± 9.2	57.1 ± 10.9	0.002
Sex (%)			0.038
Male	343(95.0)	158(90.3)	
Female	18(5.0)	17(9.7)	
cStage (%)			0.902
II	105(29.1)	50(28.6)	
III	256(70.9)	125(71.4)	
cT stage (%)			<0.001
1/2	84(23.3)	18(10.3)	
3/4	277(76.7)	157(89.7)	
cN stage (%)			0.012
0	65(18.0)	48(27.4)	
+	296(82.0)	127(72.6)	
Tumor length, cm, mean ± SD	4.9 ± 2.5	5.4 ± 2.1	0.011
Location (%)			0.021
Upper	44(12.2)	29(16.6)	
Middle	132(36.6)	68(38.9)	
Lower	90(24.9)	52(29.7)	
Unknown	95(26.3)	26(14.9)	
Differentiation (%)			0.001
Well	10(2.8)	7(4.0)	
Moderate	213(59.0)	119(68.0)	
Poor	115(3.9)	29(16.6)	
Unknown	23(6.4)	20(11.4)	
CCI (%)			0.002
0	238(65.9)	88(50.3)	
1	82(22.7)	62(35.4)	
≥2	41(11.4)	25(14.3)	
pT stage (%)			<0.001
Tis	4(1.1)	4 (2.3)	
T1	51(14.1)	6 (3.4)	
T2	60(16.6)	9(5.1)	
T3	221(61.2)	138(78.9)	
T4	25(6.9)	18(10.3)	
pN stage (%)			0.314
N0	142(39.3)	67(38.3)	
N1	108(29.9)	63(36.0)	
N2	88(24.4)	32(18.3)	
N3	23(6.4)	13(7.4)	
Margin status (%)			0.162
Negative (R0)	301(83.4)	139(79.4)	
Positive (R1/2)	56(15.5)	36(20.6)	

CRT: chemoradiotherapy; SD: standard deviation; CCI: Charlson’s comorbidity index.

**Figure 2:**
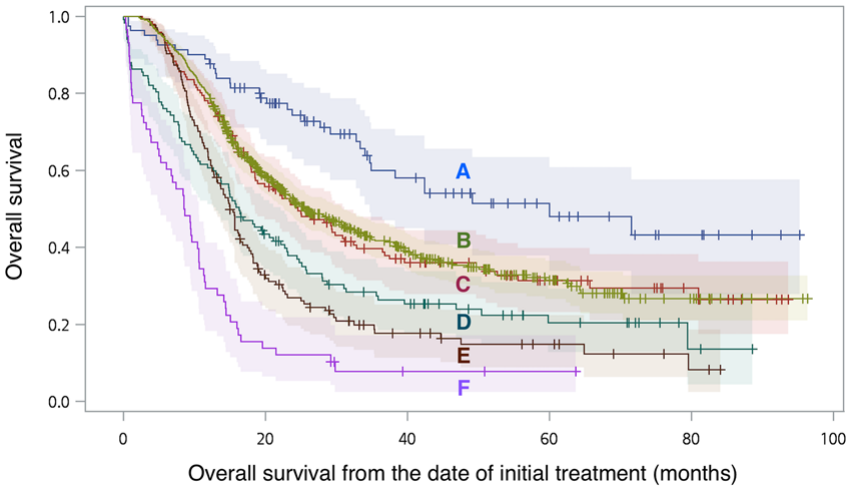
Overall survival (and 95%CI) of patients with “T1-2N0 tumors without adjuvant chemoradiation” (line A), neoadjuvant chemoradiation followed by surgery group (line B), “relatively good prognosis advanced cancer with (line C) or without (line D) adjuvant chemoradiation” and the “most advanced cancer with (line E) or without (line F) adjuvant chemoradiation”, in propensity score matched patients.

In the upfront surgery group, patients were also classified based on pathological stage and the use of adjuvant chemoradiation (CRT), namely, “T1-2N0 tumors without adjuvant CRT”, “relatively good prognosis advanced cancer with or without adjuvant CRT” and the “most advanced cancer with or without adjuvant CRT”. Whereas the “relatively good prognosis advanced cancer” included T3N0 and T1-3N1 stages, the “most advanced cancer” referred to T4, N2-3, and M1 stages. In the survival curve analysis, patients with T1-2N0 tumors obviously had the best overall survival (line A in Fig. 2). In patients with pathological stage higher than T1-2N0 stage, adjuvant CRT significantly enhanced overall survival (line C vs. D, *p* = 0.005 and E vs. F, *p* < 0.001 in Fig. 2). Moreover, in patients with “relatively good prognosis advanced cancer” who received adjuvant CRT, the overall survival was similar to that in the neoadjuvant chemoradiation followed by surgery group (line B vs. C in Fig. 2, *p* = 0.803). These observations lead to our next question: the impact of different treatment strategies on clinical T3N0 and T1-3N1 tumors.

### Analysis of clinical T3N0 and T1-3N1 population

**Figure 3:**
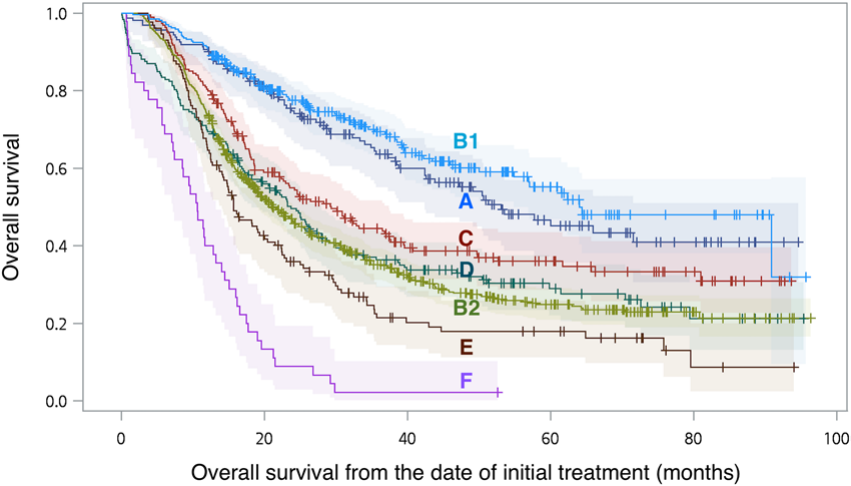
Overall survival (and 95%CI) of patients with “T1-2N0 tumors without adjuvant chemoradiation” (line A), neoadjuvant chemoradiation followed by surgery group with (line B1) or without (line B2) pathological complete response, “relatively good prognosis advanced cancer with (line C) or without (line D) adjuvant chemoradiation” and the “most advanced cancer with (line E) or without (line F) adjuvant chemoradiation”, in clinical T3N0 and T1-3N1 population.

Based on previous observations of patients with pathological T3N0 and T1-3N1 tumors, 1606 patients with clinical T3N0 and T1-3N1 tumors, including 883 and 723 in groups A and B, respectively, were identified. Patients in group A were further classified as pathological complete response (pCR, ypT0N0, n = 265) or non-pCR (n = 618). According to pathological stage and the use of adjuvant chemoradiation, patients in group B were further classified as T1-2N0 tumors without adjuvant CRT (n = 161)”, “relatively good prognosis advanced cancer with (n = 194) or without (n = 193) adjuvant CRT” and the “most advanced cancer with (n = 130) or without (n = 45) adjuvant CRT”. The first observation in the survival curves analysis (Fig. 3) is that the overall survival in patients with pT1-2N0 tumors after upfront surgery was comparable to that in patients with pCR after neoadjuvant chemoradiation (line A vs. B1 in Fig. 3, *p* = 0.301). In other words, 22.3% (161/723) of patients with clinical T3N0 and T1-3N1 tumors were found to have pathological T1-2N0 tumors. The upfront surgery strategy avoids unnecessary chemoradiation in these patients. In addition, the overall survival in patients with non-pCR after neoadjuvant chemoradiation was similar to that in patients with relatively good prognosis advanced cancer and receiving no adjuvant CRT (line B2 vs. D in Fig. 3, *p* = 0.783), but worse than that in those with adjuvant CRT (line B2 vs. C in Fig. 3, *p* = 0.010). In our cohort, 53.5% (387/723) of patients with clinical T3N0 and T1-3N1 tumors had similar pathological stages, and their survival, with the use of adjuvant CRT, was non-inferior to the neoadjuvant chemoradiation protocol. Finally, 24.2% (175/723) of patients with clinical T3N0 and T1-3N1 tumors were actually with the most advanced cancer, which means pT4, pN2-3, and pM1 stages. Even with adjuvant CRT, the outcome was very poor after upfront surgery (line E and F in Fig. 3). The accuracy of clinical staging should be improved to prevent upfront surgery in these patients.

## Discussion

Multidisciplinary therapy has been developed for esophageal cancer. Based on the results of positive trials, neoadjuvant chemotherapy and chemoradiotherapy followed by esophagectomy have become the standard treatments for esophageal cancer^1,2^. In this Taiwan Caner Registry database study, the neoadjuvant chemoradiation to upfront surgery ratio steadily increased from 0.66 in 2008 to 2.08 in 2014. Despite the advantages of early systemic micrometastasis control and tumor downstaging, neoadjuvant therapy has been criticized that the indication for chemoradiation is based on clinical staging rather than more accurate pathological staging and thus the risk of overtreatment^4^. On the other hand, upfront surgery plus adjuvant therapy is another approach of multidisciplinary treatments. The phase II trial by the Cleveland Clinic group has shown that adjuvant concurrent chemoradiotherapy has acceptable toxicity for patients with poor prognosis esophageal and gastroesophageal junction cancer^9^. Their results indicated that adjuvant treatments can be successful and may have significant advantages for clinically understaged patients or for patients with locoregionally advanced but resectable disease who undergo upfront surgical resection. Although its efficacy has not been demonstrated in a randomized controlled trial, several retrospective studies have reported that adjuvant chemoradiation can prolong overall survival and recurrence-free survival among patients with esophageal cancer and poor prognostic factors. For example, Chen *et al*. reported that adjuvant chemoradiation was effective at increasing overall survival and decreasing recurrence in lymph node-positive thoracic esophageal squamous cell carcinoma^10^. Wang *et al*. also demonstrated that adjuvant chemoradiation improved overall and progression-free survival and had significantly fewer recurrences, especially regional recurrence, in esophageal squamous cell cancer with extracapsular lymph node extension^11^.

According to the analysis of the Taiwan Cancer Registry database, we have previously suggested pT3/4 stage, positive lymph node involvement, larger tumor size, poorly differentiated tumors, and R1/2 resection as indications for adjuvant chemoradiation^6^. We also showed that surgery followed by adjuvant chemoradiation was significantly more effective than surgery alone at increasing the overall survival and decreasing recurrences, especially locoregional recurrences^7^. Moreover, our recent report demonstrated that the outcome after surgery and adjuvant chemoradiation could be similar to that after neoadjuvant chemoradiation followed esophagectomy. For patients who completed trimodal treatments, including chemoradiotherapy and esophagectomy, there was no difference in overall survival or disease-free survival between the neoadjuvant (preoperative) and adjuvant (postoperative) chemoradiation groups^8^. All these observations motivate us to evaluate the impact of “upfront surgery and pathological stage-based adjuvant chemoradiation” strategy, which means the indication for chemoradiotherapy is based on pathological stage after upfront esophagectomy. Whereas no further treatments for pathological T1-2N0 tumors, patients with poor prognostic factors, i.e., pathological stage higher than T1-2N0 tumors, would receive adjuvant chemoradiation. We found that a significant portion of clinical stage II/III patients were actually with pathological stage I (T1-2N0), in which unnecessary chemoradiotherapy could be avoided with an upfront surgery strategy. In both original and matched cohorts, the survival of the “upfront surgery and pathological stage-based adjuvant chemoradiation” group was not inferior to that of the “neoadjuvant chemoradiation followed by surgery” group. Our findings echo with reports in the literature. In a retrospective study of patients with clinical stage II or higher esophageal adenocarcinoma, minimally invasive esophagectomy was performed regardless of tumor stage or the use of neoadjuvant therapy. Guided by pathological stage, adjuvant treatment was administered to 49% of upfront esophagectomy patients. The authors reported that primary minimally invasive esophagectomy followed by adjuvant chemotherapy does not negatively influence survival compared with patients treated with neoadjuvant therapy^4^. In another study of the multimodal treatment combination of upfront surgery followed by adjuvant chemotherapy for esophageal squamous cell carcinoma, 45% of patients with positive lymph node involvement received adjuvant treatment, and the completion rate was as high as 91%. Patients who received adjuvant chemotherapy showed longer survival than those who underwent surgery alone. However, no significant difference in overall survival was observed between the neoadjuvant treatment and upfront surgery groups^5^. In contrast, in the pivotal CROSS study, no further treatment was administered to patients in the surgery alone group, even as high as 75% of patients had pathologically positive lymph node metastasis^3^. To some extent, the CROSS study compared the neoadjuvant chemoradiation group to an undertreated group. A randomized controlled trial comparing different combinations of multimodal treatments is justified.

Although the “upfront surgery and pathological stage-based adjuvant chemoradiation” strategy seems to be a reasonable approach in our study, there are several caveats. First, the indications for adjuvant chemoradiation were not randomized in our study. Whereas some hospitals in Taiwan recommend adjuvant chemoradiation for patients with pathologically poor prognostic factors, some stick to the NCCN guideline and just suggest surveillance as long as there is no residual tumor^12^. Additionally, patients who underwent surgery only without adjuvant treatment had a higher age and CCI compared to those who underwent adjuvant chemoradiation in our study, implying that patient age and comorbidities are critical reasons that interfere with the decision for adjuvant chemoradiation. Second, in the analysis based on pathological stage, patients who received upfront surgery but had the “most advanced cancer,” i.e., the most advanced cancer. The pT4, pN2-3, and pM1 stages were associated with dismal prognosis despite the use of adjuvant chemoradiation. Compared to pT3N0 and pT1-3N1 stages, which have limited lymph node involvement and no adjacent organ invasion, the most advanced cancers are at high risk for incomplete resection; thus, they should be avoided in the upfront surgery strategy. In the current study, nearly one-fourth of patients with clinical T3N0 and T1-3N1 tumors actually had the most advanced cancer. The accuracy of clinical staging should be improved to decrease the proportion of clinically under-staged patients.

The Taiwan Cancer Registry database has standardized definitions of terminology, coding and procedures of the registry’s reporting system. There are also several corrigendum procedures applied to ensure the completeness and accuracy of cancer registration data, e.g., hospitals are required to carry out a self-check procedure using standardized logic algorithms and software provided by the central office to identify and correct potential errors before the data submission^13^. In this study, pretreatment tumor factors and patient comorbidities were included in the propensity score matching to decrease potential bias caused by factors that may interfere with treatment decisions. However, we have no data regarding neoadjuvant chemoradiation-related toxicities and surgical complications. Patients who failed to survive neoadjuvant chemoradiotherapy were not included. Similarly, patients who failed to survive upfront surgery and could not receive adjuvant chemoradiation were categorized in the upfront surgery only group. In addition, the toxicity of adjuvant chemoradiation was not evaluated in this study. However, it has been shown that the completion rates of adjuvant therapy are between 65 and 91%^4,5,10,14^. In our previous study, less than 10% of patients in the adjuvant chemoradiation group received radiation doses less than 40 Gy, suggesting that most patients could complete the entire course of adjuvant treatments^6^. Indeed, it has been adopted that early recovery after minimally invasive esophagectomy will improve the delivery of adjuvant therapy, whose role needs to be reappraised in the era of minimally invasive surgery. Furthermore, the Taiwan Cancer Registry database lacks detailed information on staging workup studies, chemoradiation regimens, radiation fields, and surgical techniques, which constitute the limitations of this study.

In conclusion, there was no significant difference in overall survival between the standard neoadjuvant chemoradiation followed by surgery protocol and the “upfront surgery and pathological stage-based adjuvant chemoradiation” strategy, in which adjuvant chemoradiation was administered to patients with poor prognostic pathological factors. However, patients with higher age and comorbidity scores may not be able to complete adjuvant treatments. In addition, patients with pathological T4, N2-3, and M1 had very poor outcomes even after adjuvant chemoradiation. Therefore, patient selection and accurate clinical staging are prerequisites when adopting upfront surgery strategies.

## Methods

This study was carried out in accordance with the relevant guidelines and regulations. The Institutional Review Board of Taipei-Veterans General Hospital approved this study and granted a waiver of the informed consent process (IRB_2015-06-001BC). Data source acquisition was as described previously^6–8^. In brief, patient data were obtained from the Taiwan Cancer Registry database, which is a national population-based database organized by the Health Promotion Administration, Ministry of Health and Welfare (MOHW), Taiwan. Confidentiality was ensured by the Health and Welfare Data Science Center (HWDC), MOHW, Taiwan, which encrypted individual identifiers to protect privacy before releasing information to investigators for research purposes. The diagnosis was based on their International Classification of Diseases for Oncology (ICD-O-3) site codes (C15.0–C15.5, C15.8, and C15.9) and morphology codes (8070–8076, and 8083). The staging results were determined according to the seventh edition of the American Joint Committee on Cancer TNM classification system. The inclusion criteria for this study were patients with clinical stage II and III ESCC diagnosed between 2008 and 2014 who had undergone neoadjuvant chemoradiation followed by surgical resection (group A) or upfront esophagectomy (alone or with adjuvant chemoradiation, group B) as the initial treatment modalities. The exclusion criteria included incomplete clinicopathological information, which precluded statistical analysis. Individual patient-level data were linked with the National Register of Deaths Database for survival status confirmation and the date of death and the National Health Insurance database for comorbidities identification. Charlson’s comorbidity index (CCI) was calculated using ICD-9-CM codes, excluding cancer-related disease, in the year before starting initial treatment^15^.

### Statistics

Categorical and continuous variables were compared with the chi-square test and Student’s t-test, respectively. Propensity score matching was performed to decrease confounding effects due to nonrandomized assignment. First, a propensity score for each patient was calculated by logistic regression using the variables of age, sex, clinical stage, tumor location, differentiation grade, tumor length, and comorbidity score. Then, a 1:1 matched study group was created using a greedy matching algorithm. After matching, 562 well-balanced pairs of patients in groups A and B, respectively, were identified for outcome comparison. Univariable and multivariable survival analyses were analyzed using the Cox proportional hazards regression model. Survival curves were plotted using the Kaplan–Meier method and compared with the log-rank test. The overall survival was calculated as the period between the date of initial treatment and the date of death. Patients who survived to the end of the follow-up period (December 31, 2015) were censored. All statistical calculations were performed with Statistical Analysis System (version 9.3; SAS Institute, Inc., Cary, NC) and Statistical Product and Service Solutions (version 20; SPSS Inc., Chicago, IL). A *p* value < 0.05 was considered statistically significant.

## Electronic supplementary material

Patient enrollment
