# Supplementary material for: Upfront surgery and pathological stage-based adjuvant chemoradiation strategy in locally advanced esophageal squamous cell carcinoma
Source: Sci Rep. 2018 Feb 1;8:2180. doi: 10.1038/s41598-018-20654-0 (PMC5794775; doi:10.1038/s41598-018-20654-0)

Upfront surgery and pathological stage-based adjuvant chemoradiation strategy in locally advanced esophageal squamous cell carcinoma

Hui-Shan Chen, Po-Kuei Hsu, MD, Chia-Chuan Liu, Shiao-Chi Wu

Figure S1. Patient enrollment

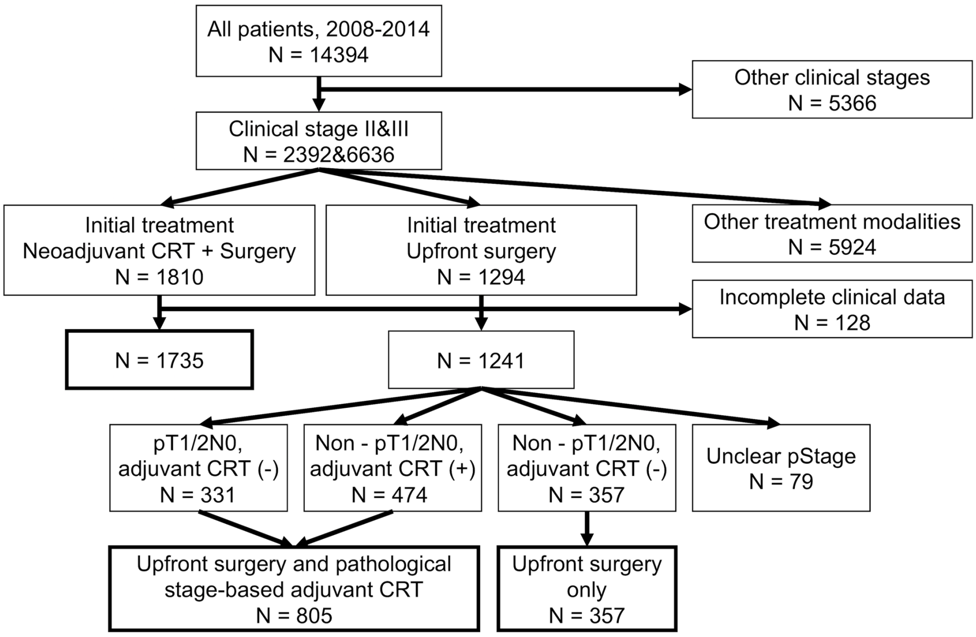

Supplement: Supplementary file 1 — Patient enrollment [file 41598_2018_20654_MOESM1_ESM.pdf]
